# A comparison of knowledge and attitude toward mental illness among secondary school students and teachers

**DOI:** 10.1186/s40359-022-00820-w

**Published:** 2022-04-29

**Authors:** Omar Al Omari, Atika Khalaf, Iman Al Hashmi, Mohammad Al Qadire, Yousef Abu Shindi, Sulaiman Al Sabei, Nasir Matani, Devanprabudoss Jesudoss

**Affiliations:** 1 College of Nursing, Sultan Qaboos University, Muscat, Oman; 2 Faculty of Health Science, Kristianstad University, 291 88 Kristianstad, Sweden; 3 Faculty of Nursing, Al Al-Bayt University, Mafraq, Jordan; 4 College of Education, Sultan Qaboos University, Muscat, Oman

**Keywords:** Knowledge, Attitude, Secondary school students, Secondary school teachers, Adolescents

## Abstract

**Aim:**

The current study aimed to assess and compare the level of knowledge and attitude towards mental illness between secondary school students and their teachers in Oman.

**Methods:**

An online survey was carried out to collect data from 400 students and 411 teachers about their knowledge and attitudes toward people with mental illness. Two independent case studies about depression and schizophrenia were also tested.

**Results:**

Students have a poor knowledge of mental illness compared with their teachers, as more than half scored less than 60% compared with 16.5% of the teachers. More than two-thirds of the students (80%) and teachers (76.4%) have a low or minor positive attitude toward people with mental illness. The study identified significant differences in knowledge in favour of teachers, although the opposite was found regarding attitudes.

**Conclusions:**

Since students spend a significant amount of time in school, bridging the gap between teachers' and students’ knowledge and attitudes toward mental illness is an essential part in enhancing the knowledge and attitudes of the students. In addition, knowledgeable teachers with positive attitude can assist in early identification of mental illnesses and help students when needed. In turn, students who possess knowledge and positive attitude toward mental illness can share their concerns with their teachers. In the presence of such accepting and cooperative environment, the stigma can be decreased and early detection of mental illness and help-seeking behaviour can be promoted.

## Introduction

The prevalence of mental health disorders has steadily increased globally over time, with almost one-fifth of adolescents suffering from a serious mental illness [1]. In fact, the majority of mental illnesses emerges before the age of 18 [2, 3]. Untreated mental illness among secondary school students can lead to social, behavioural, and academic issues, as well as deteriorating symptoms or impairment, other health comorbidities, suicide-related behaviours, and the onset of chronic disease in adulthood [4–6]. The public hold negative attitudes toward people with mental illness, including believing that they are dangerous, incompetent, unpredictable, weak, and unable to complete given duties [7]. As a result, sufferers are alienated, isolated, and excluded from the larger community. Secondary and university students between the ages of 15 and 24 years have been shown to have similar negative attitudes toward people with mental illness as adults [8, 9]. Although the general public’s knowledge about mental illness has improved, a negative attitude among Jordanian adolescents remains [8, 10]. Gender, level of education, previous mental health training, having a family history with a mental illness, and/or socio-economic status were found to predict stigma among teachers and their students [11, 12].

Since nearly half of all mental disorders have evolved by the middle of adolescence, schools play a crucial role in providing instruction to students and raising their awareness about people with mental illness [13]. In fact, one of the modern responsibilities of teachers is to help and support students’ mental health needs [14–16]. Teachers can help in the early detection of mental health problems faced by their students [16], and coordinate with school counsellors, parents, and other mental-health experts [17]. They can also answer students’ questions, reduce stigma about mental illness, and encourage students to seek help [18]. Therefore, the knowledge and attitudes of teachers about mental illness are important components to study and ensure that no shortage that can compromise these important roles is occurring [19]. Previous studies showed that Chines, Indian, and Kenyan teachers have poor knowledge about the signs of mental disease, prevalence, causes, and treatment [20–22]; hence, they were unable to detect early warning signs of mental illness in their students. However, interventional studies have shown a significant improvement in students’ mental wellbeing [23], with students reporting that they receive more information about mental health from teachers who have attended mental health programmes [24]. Improving teachers’ mental health literacy may lead to increased access to appropriate services for students, reducing the negative consequences of undiagnosed and untreated mental disorders in children and adolescents [22, 25]. However, exploring mental illness among students and their teachers in the context of the school environment has received insufficient attention from researchers in developing countries [22]. The aim of the current study was therefore to assess and compare the level of knowledge and attitude toward mental illness among secondary school students and their teachers in Oman. Comparing students and teachers may help in identifying possible significant differences and thus pave the way for a more health promoting, bridging the differences, and enhancing both groups’ knowledge and attitude approach. This in turn will assist in creating a positive environment where early detection and support to students who are in need can be improved. Hence, the research null hypotheses are; there is no significant difference between the students and their teachers’ total knowledge scores about mental illness; and there is no significant difference in the total attitude scores of teachers compared with their students toward people with mental illness.

## Methods

### Design

The researchers adopted a cross‐sectional research design with self-administered questionnaires.

### Sample

The researchers recruited adolescents between the ages of 15 and 19 and their teachers, registered in government schools, using a stratified random sampling technique. Government schools were chosen to ensure a population from various socio-economic backgrounds, to promote the generalizability of the study’s findings. There are 56,385 Omani teachers and 216,747 Omani students of the defined age [26]. The researchers calculated sample size using Slovin’s formula [n = N/[1 + N e^2^], where n = number of participants, N = total population and e = margin of error, [0.05]. Based on the equation, 399 school students and 398 teachers required to participate in the study.

### Ethical considerations

Prior to data collection, ethical approval was obtained from the Institutional Review Board in the College of Nursing at Sultan Qaboos University [CON/EA/21/2019] and the Omani Ministry of Education. The Helsinki Declaration (1964) and its later amendments guided all performed procedures. Informed consent occurred prior to participation in the study. Additional guardian consent occurred for students under the age of 16. Researchers informed participants that their participation is entirely voluntary, and that they could drop out at any time or choose not to answer specific questions. Participants’ confidentiality was protected as no personal information was obtained, and the findings could only be published in aggregate.

### Data collection

The researcher collected the data using self-reported questionnaires between March and the end of May 2021. They generated a list of 67 schools located in Muscat, Batinah, and Sharqiyah governorates, and 14 schools (7 male and 7 female) were randomly selected to recruit students. The whole classes asked to participate to ensure an equal distribution of age groups and cisgender. For the teachers, the selection was expanded to 15 male and 15 female randomly selected schools. The entire teaching staff asked to participate to ensure the equal distribution of age groups and cisgender. These chosen governorates purposefully selected because they include the largest number of students. The researchers sent a link containing an information sheet, consent form, a demographic data form, and three self-administered questionnaires to the teachers and parents through the school administration. Teachers, parents, and students asked to read the information sheet and contact the principal investigator to answer any questions. If the teachers consented, they completed the surveys themselves; if parents consented, the children completed the survey independently.

### Measures

The researchers used a structured questionnaire to collect information about the following: (1) participants’ socio-demographic data, including age, cisgender, education level, family income, marital status, and parents’ level of education, self-reported diagnosis of mental illness, presence of family member or friend diagnosed with a mental illness, and if they had taken a course about mental illness; (2) knowledge about mental illness questionnaire; (3) case assessment; and (4) attitude toward mental illness questionnaire.

#### Knowledge about mental illness

The knowledge about mental illness was measured using a 20-item survey based on the “Core Information and Knowledge Essentials of Mental Health Publicity and Education” developed by the Chinese Ministry of Health [27] to assess public knowledge. “Mental health is a component of health” is an example of the items used. A correct Yes/No answer to items 1–16 scored one point and a wrong answer zero. Positive responses to questions 17–20, “know”, generated one point, and “don’t know” zero. The higher the score, the better the mental health knowledge. To make the knowledge test grade more meaningful, the grade recorded in Table 1 was transformed into a 100-grade points system, with the pass score set at 60, reflecting the lower pass limit practiced in the Omani educational system. The internal reliability was established: Cronbach’s alpha 0.73 in the previous analysis [28], and 0.72 in our study. With regards to the tool’s validity, a Confirmatory Factor Analysis (CFA) using AMOS v.22.0 to examine the one-factor structure of the knowledge about mental illness scale results indicated an acceptable data fit (χ^2^ = 893.81, *p* > 0.001; CFI = 0.88; GFI = 0.90; RMSEA = 0.07).

**Table 1:** Participants’ characteristics (teachers N = 411 & students N = 400)

Variable	Students		Variable	Teachers	
	n	%		n	%
Cisgender			Cisgender		
Male	165	41.3	Male	151	36.7
Female	235	58.8	Female	260	63.3
Monthly family income (OMR)			Monthly income (OMR)		
Less than 500	142	35.5	Less than 500	0	0
500–999	156	39.0	500–999	212	51.6
1000–1499	64	16.0	1000–1499	139	33.8
1500–2000	38	9.5	1500–2000	60	14.6
Self-report of mental illness			Self-report of mental illness		
No	394	98.5	No	400	97.3
Yes	6	1.5	Yes	11	2.7
Family member with a mental illness			Family member with a mental illness		
No	379	94.8	No	378	92
Yes	21	5.3	Yes	33	8
Friend with a mental illness			Friend with a mental illness		
No	389	97.3	No	356	86.6
Yes	11	2.8	Yes	55	13.4
Had a course about mental illness			Had a course about mental illness		
No	328	82	No	384	93.4
Yes	72	18	Yes	27	6.6
Knowledge related to mental illness			Knowledge related to mental illness		
< 60	216	54.0	< 60	68	16.5
60–69	94	23.5	60–69	115	28.0
70–79	51	12.8	70–79	130	31.6
80–89	16	4.0	80–89	61	14.8
90–100	23	5.8	90–100	37	9.0
Positive attitude			Positive attitude		
Low	11	2.8	Low	28	6.8
Minor	148	37	Minor	187	45.5
Moderate	172	43	Moderate	127	30.9
High	69	17.3	High	69	16.8

	m	SD		m	SD
Age	16.9	0.95	Age	33.1	6.9
Knowledge scale	10.5	4.0	Knowledge scale	13.7	2.2
Positive attitudes	36.8	6.4	Positive attitudes	34.8	6.24

#### Case assessment questionnaire (CAQ)

The Case Assessment Questionnaire (CAQ) was developed by the Chinese Ministry of Health [27]; it has five cases: depression, schizophrenia with positive symptoms, mania, schizophrenia with negative symptoms, and obsessive–compulsive disorder. The first two cases are the most significant and must be examined; if the situation allows, all five cases may be tested. In all five cases, items 1 and 2 were knowledge questions, while questions 3 to 9 were related to attitude and beliefs; no standard answers were given. The reference answers for the first case’s questions 1 and 2 are 3 and 3, and for the second case’s questions 1 and 2 are 3 and 6. The current researchers used the first two cases to further assess the knowledge and attitude of the participants with regards to these common mental health illnesses.

#### Attitude questionnaire

The attitude questionnaire is a 12-item survey developed by the Chinese Ministry of Health [27] to measure the public attitude to mental illness. The items are formulated as statements in which the participants are asked to agree or disagree (five possible responses), e.g. “Most people believe that people who have been in a mental hospital have the same intelligence as ordinary people”. For items 1, 2, 3, 4, 8, and 10: “Completely agree” receives 5 points, “basically agree” 4 points, “hard to say” 3 points, “basically disagree” 2 points, and “not at all” or “disagree” 1 point. Items 5, 6, 7, 9, 11, and 12 were reversed. Finally, the overall score for the mental illness-related attitude questionnaire was calculated, with possible total scores ranging from 12 to 60. The higher the total score, the more positive attitude towards mental illness. Based on the current sample distribution, the standardized z scores were calculated for students’ and teachers’ total scores, then cut-off scores were determined, based on *p* = 0.05 (± 1.96); (< = − 1.96) points was regarded as low; (− 1.961–0) as mild; (0.111–1.96) as moderate, and (> 1.96) as high. In this analysis, Cronbach’s alpha was 0.98. To test the tool’s validity, CFA was conducted to examine the one-factor structure of the positive attitude questionnaire scale, the results indicating an acceptable data fit (χ^2^ = 281.05, *p* > 0.001; CFI = 0.92; GFI = 0.95; RMSEA = 0.072).

### Data analysis plan

Data was downloaded from Google Forms and exported first to an Excel sheet and then to SPSS version 23. Frequencies, mean, and standard deviation were used to describe the participants’ demographics. Since the dependent variables, knowledge about and positive attitudes toward mental illness, were not normally distributed, the non-parametric tests of Mann–Whitney U-test and Kruskal–Wallis were used to test the differences in means between teachers and students. Spearman correlation was also used to test the correlation between knowledge about mental illness and positive attitudes toward it. No missing data were found. An alpha-level of *p* < 0.05 was set for significance in all analyses.

## Results

### Demographics

Four hundred students with an average age of 16.9 years (SD = 0.95) completed the survey; almost two-fifths were male, just over a third had a family income less than 500 Omani Ryals, and just under a fifth had attended a course on mental illness. On the other hand, 411 teachers with an average age of 33.1 years (SD = 6.9) completed the survey; almost two-thirds (63.3%) were female, an almost half had a monthly salary ranging between 500 and 999 Omani ryals. Most teachers (97.3%) and their families (92%) did not have a mental illness. 54% and 16.5% of students and teachers score less than 60 in the knowledge test. Only 17.3% and 16.8% of the students and teachers had a high positive attitude toward people with mental illness. For more detail, see Table 1.

### Bivariate analysis of knowledge and attitude based on selected demographics for teachers and students

The knowledge about mental illness was greater among female teachers (Mdn = 14) than males (Mdn = 13), *p* < 0.01. It was also greater for teachers who had a family member with mental illness (Mdn = 15, *p* = 0.014); or friend with mental illness (Mdn = 15, *p* = 0.015). Teachers with no family member with a mental illness had a more positive attitude toward people with mental illness (Mdn = 35, *p* = 0.014). Knowledge of mental illness was greater among male students (Mdn = 12) than females (Mdn = 10), *p* < 0.001; and among students who had attended a course on mental illness (Mdn = 13, *p* < 0.01). However, students who had a family member with mental illness (Mdn = 39, *p* = 0.028); and had taken a course about mental illness (Mdn = 38, *p* = 0.018) had significantly higher scores of positive attitudes. See Table 2 for more detail.

**Table 2:** Bivariate analysis of knowledge and attitude with respect to teachers’ and students’ characteristics

Variable	Teachers				Students			
	Knowledge		Attitude		Knowledge		Attitude	
	Mean Rank	*p* value	Mean Rank	*p* value	Mean Rank	*p* value	Mean Rank	*p* value
Cisgender		< 0.01		0.886		< 0.01		0.484
Male	171.11		207.10		222.82		195.69	
Female	226.26		205.36		184.83		203.88	
Monthly family income (OMR)		< 0.01		< 0.01		0.947		0.003
Less than 500	0		0		199.17		229.67	
500–999	205.72		224.63		177.19		165.70	
1000–1499	208.08		191.36		220.43		183.34	
1500–2000	202.18		174.12		267.61		263.26	
Self-report of mental illness		0.211		0.134		0.018		0.740
No	204.80		204.45		202.18		200.74	
Yes	249.82		153.27		89.92		185.0	
Family member with a mental illness		0.014		0.004		0.532		0.028
No	201.78		210.92		201.35		197.52	
Yes	254.35		149.64		185.21		254.31	
Friend with a mental illness		0.015		0.713		0.011		0.584
No	200.47		205.15		202.96		201.03	
Yes	241.81		211.47		113.5		181.73	
Had a course about mental illness		0.447		0.648		< 0.01		0.018
No	204.83		205.29		185.44		194.14	
Yes	222.67		216.06		296.10		229.47	

### The mean difference in knowledge and attitude between students and teachers

A Mann–Whitney test indicated that knowledge of mental illness was greater among teachers (Mdn = 14) than students (Mdn = 11), *U (N*_*teachers*_ = *411, N*_*students*_ = *400)* = *41,250*, z = − 12.335, *p* < 0.01. However, positive attitudes toward people with mental illness was greater for students (Mdn = 36) than for teachers (Mdn = 35), *U (N*_*teachers*_ = *411, N*_*students*_ = *400)* = *68,463*, z = − 4.130, *p* < 0.01.

### Association between age, knowledge, and attitude toward people with mental illness

A Spearman’s test indicated a significant weak negative correlation between the teachers’ knowledge and a positive attitude toward mental illness, r_s_ = − 0.178, *p* < 0.01, *N* = 411. However, there was a significant weak positive correlation between the students’ knowledge and a positive attitude toward mental illness, r_s_ = 0.249, *p* < 0.01, *N* = 400.

### Differences between students’ and teachers’ responses based on the two selected cases

Table 3 shows that significantly more teachers (totally 92.2%) than students [totally 85%] were unable to identify the nature of the depression problem (mental problem). However, the correct diagnosis [depression] was identified by significantly more teachers (59.1%) than students (34.0%). However, in the second case, schizophrenia with positive symptoms, 37% and 50.1% of students and teachers respectively were able to identify the case as a mental illness, although only 7.8% and 14.6% of students and teachers were able to give correct diagnoses. Interestingly, 21.5% of the students and 12.2% of the teachers believed that the problem was related to possession by spirits. In both cases, participants disagreed about what to recommend. For example, in the case of depression, 16.3% of the students and 27.7% of the teachers advised talking to a friend and family. Only 16.5% and 19% respectively recommended seeing a psychiatrist and only 17.9% of the total population recommend visiting a GP. For more information see Table 3.

**Table 3:** The differences in responses between teachers and students in regard to two selected cases

	Depression					Schizophrenia with positive symptoms				
	Students		Teachers		*p* value	Students		Teachers		*p* value
	n	%	n	%		n	%	n	%	
What type of problem is this?					< 0.01					< 0.01
Burnout	241	60.3	332	80.8		84	21.0	116	28.2	
Physical problems	46	11.5	47	11.4		27	6.8	39	9.5	
Mental problems	60	15.0	32	7.8		148	37.0	206	50.1	
Possession by spirits	8	2.0	0	0		86	21.5	50	12.2	
None of above	45	11.3	0	0		55	13.8	0	0	
What is the name of this type of problem?					< 0.01					< 0.01
Poor physical health	82	20.5	33	8.0		63	15.8	15	3.6	
Neurasthenia	45	11.3	25	6.1		29	7.2	41	10.0	
Depression	136	34.0	243	59.1		50	12.5	65	15.8	
Mania	13	3.3	3	0.7		64	16.0	56	13.6	
Obsessive–compulsive disorder	12	3.0	20	4.9		76	19.0	100	24.3	
Schizophrenia	6	1.5	8	1.9		31	7.8	60	14.6	
Other problems	12	3.0	27	6.6		27	6.8	24	5.8	
Do not know	94	23.5	52	12.7		60	15.0	50	12.2	
What is the main cause of this type of problem?					< 0.01					< 0.01
Negative life events	142	35.5	148	36.0		112	28.0	137	33.3	
Work stress	106	26.5	109	26.5		40	10.0	31	7.5	
Hereditary illness	10	2.5	9	2.2		22	5.5	13	3.2	
Problem with thinking	58	14.5	100	24.3		94	23.5	143	34.8	
Possession by spirits	22	5.5	6	1.5		69	17.3	35	8.5	
Other causes	62	15.5	39	9.5		63	15.8	52	12.7	
What would you recommend this person do?					< 0.01					< 0.01
Talk to friends and family	65	16.3	114	27.7		86	21.5	88	21.4	
Relax more	145	36.3	137	33.3		55	13.8	24	5.8	
See GP	49	12.3	23	5.6		21	5.3	19	4.6	
Traditional medicine	6	1.5	8	1.9		17	4.3	19	4.6	
See a psychiatrist	68	17	110	26.8		135	33.8	231	56.2	
Other	17	4.3	17	4.1		26	6.5	30	7.3	
Don’t know	50	12.5	114	27.7		60	15.0	88	21.4	
How would most neighbours view this person?					< 0.01					< 0.01
Think the individual is strange	117	29.3	50	12.2		89	22.3	99	24.1	
Fear person’s behaviour	45	11.3	42	10.2		196	49.0	154	37.5	
Sympathize	121	30.3	252	61.3		24	6.0	92	22.4	
Considered normal	50	12.5	67	16.3		32	8	28	6.8	
None of the above	67	16.8	0	0		59	14.8	0	0	
Do you think it is possible that you would ever be like this?					< 0.01					< 0.01
Yes	203	50.7	285	69.3		88	22.0	144	35.0	
No	179	49.3	126	30.7		312	79	267	65.0	
Is the individual able to tell right from wrong?					< 0.01					< 0.01
Yes	133	33.3	201	48.9		83	20.8	127	30.9	
No	76	19.0	54	13.1		143	35.8	140	34.1	
Hard to tell	191	47.8	156	38.0		174	43.5	0	0	
Is the individual able to continue to work?					< 0.01					< 0.01
Yes	90	22.5	64	15.6		87	21.8	62	15.1	
Yes, after resting	101	25.3	194	47.2		48	12	62	15.1	
Yes, after treatment	121	30.3	129	31.4		165	41.3	250	60.8	
No	88	22.0	24	5.8		100	25.0	37	9.0	
Is the individual’s risk of harming others increased?					< 0.01					0.006
Yes	131	32.8	106	25.8		179	44.8	221	53.8	
No	243	60.8	305	74.2		221	55.3	190	46.2	

## Discussion

The aim of the current study was to assess and compare the level of knowledge and attitude toward mental illness among secondary school students and their teachers in Oman. An interesting finding is the significant positive association between knowledge and positive attitudes among students and the weak negative association between these variables among teachers. One possible explanation is that our sample of students is young and might not have been exposed to the negative influence of culture and mass media [29]. Nevertheless, their knowledge may be the source of improvement in their attitude, since those who had taken a course on mental illness had more positive attitudes. However, several other factors may explain teachers’ knowledge and attitude; for example, previous experience, mass media and culture may negatively affect their attitude. Our findings allude to the importance of improving individuals’ knowledge about mental illness as early as possible, to neutralize other contributory factors. It would also be important to conduct a qualitative study to explore this finding in depth.

Overall, the total scores for knowledge and positive attitude toward people with mental illness were low in the current study. Previous research in the Arab region [8, 9, 11, 30] and elsewhere [31] had similar results among students and teachers [21, 32]. There is a need to integrate or adopt mental health programmes to refresh and improve teachers’ current knowledge about mental illness. Internationally, different programmes are available providing teachers with knowledge about depression, schizophrenia, and stress [33] and teaching them how to build self-esteem among students. The effectiveness of these programmes is well established [34, 35]; hence, decision- makers in Oman may benefit from adopting or building on these existing programmes.

In the current study, knowledge about mental illness was significantly greater among female teachers and those with a family member or friend with mental illness. Similarly, Parikh, Parikh [21] showed that secondary school teachers in general report inadequate knowledge regarding mental illness, with comparatively more female than male teachers showing greater knowledge. Female secondary school teachers seem to have low [36] or moderate [37] mental health knowledge in the region. Since the teachers’ mental health literacy is strongly linked to the students’ knowledge [38], it is essential to improve teachers’ knowledge and attitudes and in result gain an improvement of the early detection and an encouragement of students to seek professional mental health help. Teachers need to build a multi-professional school-based team to provide support to students with mental health problems [39]. Local as well as regional programmes to enhance or refresh the teachers’ knowledge need to be designed and tested for their effectiveness. Studies testing such programmes have shown significant improvement in teachers’ knowledge post-intervention, as described in different systematic reviews [40, 41]. School nurses and counsellors can play a significant role in developing and/or delivering these programmes.

The opposite of teachers was seen in students with regard to gender, namely knowledge about mental illness was significantly greater among male students in our sample. This finding might be unique to our population since other studies [31] found no differences between boys’ and girls’ knowledge level. The findings also contradict those shown in Western literature [42, 43], where adolescent girls have higher levels of mental health knowledge than boys. A comprehensive literature review investigating mental illness in the Arab world reported similar findings to the current study, namely that males and those with higher levels of education and/or a family member or a friend with mental illness showed more positive attitudes toward mental illness [29]. The findings of the current study could benefit from a discussion of the possible effects of environmental and socio-cultural factors. One possible explanation for our finding is that Arabic culture gives male adolescents more freedom to discuss sensitive matters, compared with their female counterparts. Boys and girls need to be provided with sufficient information about mental illness in supportive and secure environments like schools.

A Mann–Whitney test indicated that knowledge of mental illness was significantly greater among teachers. However, positive attitudes toward people with mental illness was significantly greater for students. Thus, both our null hypotheses are rejected. In addition, knowledge was significantly greater among students who had taken a course about mental illness. This is in line with previous research [23, 24]. Mental health programmes should be an integral part of teachers’ preparatory courses. Improving their mental health literacy may have positive consequences on the students’ knowledge and may lead to increased access to appropriate mental health services for students, and a reduction in the negative consequences of undiagnosed and untreated mental disorders in adolescents. These consequences could be poor academic and vocational performance, social instability, and early mortality due to suicide [30]. Students need similar programmes in which teachers, school nurses, and psychologists can play an integral part. These programmes should target not only knowledge but also the attitudes of teachers and students toward mental illness, to counteract the current negative attitudes and beliefs. For example, some students and teachers in the current study still believed that mental illness could be the result of possession by spirits, and that persons with mental illness are violent, and untrustworthy at work. Arab populations from several Middle Eastern countries, as well as those living in Western societies, perceive mental illness negatively, and that mass media play a significant role in this negative view [29, 44]. Teachers’ own education can have a positive impact on students and other teachers.

Overall, our results stress the importance of developing and integrating an educational programme to increase students’ and teachers’ knowledge and attitude about mental illness, as the current curriculum has no topics covering this topic. In this regard, school nurses and counsellors should play an active role and have a fixed topic covering mental illness in their daily work with students and teachers. Normalizing mental illness, correcting myths, and implementing effective interventions in society in general and in school students specifically might enhance overall knowledge and attitude about mental illness. Teachers have a significant role in this process [18] and might benefit from regular courses or workshops on how to detect symptoms and signs of mental illness among students. There is also a need for experimental studies to test the effectiveness of existing educational programmes in the Omani context.

Methodologically, most of our findings were significant, perhaps indicating a type one error. However, some of the comparison groups were smaller than others in certain statistical tests, which might explain the significant differences. However, the number of students and teachers were comparable, making the statistical analysis reliable. In addition, taking the broad sample representing students and teachers from three different governorates, the random selection of these schools, and the equal representation of male and female schools, the generalizability of the results is considered to be enhanced and of interest to the whole community of school children and teachers. Yet, considering the mass-significance, the generalizability of the findings might be limited to schools from similar context. Also, the nature of the self-reporting data might have introduced a bias related to over- or underestimation of the own knowledge and/or attitudes which in turn could limit the generalizability of the findings.

Another possible limitation of the current study is that we were unable to match the teachers with their students, which could have given an important insight and a possible explanation of the true relationship between the teachers’ and their students’ knowledge and attitudes. However, based on the ethical considerations, we weighed confidentiality as being higher and did not collect any personal data, such as the school in which the participants were based. Pairing teachers with students in future replicate or similar studies is recommended.

## Conclusion

Our study findings stress on the important role of the teacher in enhancing the knowledge and attitudes of students toward mental illness. Since students spend a significant amount of time in school, bridging the gap between teachers' and students’ knowledge and attitudes toward mental illness is an essential part in enhancing the knowledge and attitudes of the students. In addition, knowledgeable teachers with positive attitude can assist in early identification of mental illnesses and help students when needed. In turn, students who possess knowledge and positive attitude toward mental illness can share their concerns with their teachers. In the presence of such accepting and cooperative environment, the stigma can be decreased and early detection of mental illness and help-seeking behaviour can be promoted.

Given the limited studies exploring the knowledge and attitude of students and teachers in Middle Eastern countries, the current study is the first of its kind to collect data at a single point in time and to provide insight into the situation; it can be used as a reference point for both regional and international studies. It is crucial to ensure that teachers have the necessary knowledge, confidence, and skills to support and educate students about mental health. Thus, decision-makers and policy-makers might benefit from our findings and consider developing educational material, implement the education, and test its effectivity in improving the knowledge and attitudes of students and teachers.

## Data Availability

The data set associated with the paper is available from the Principal Investigator, Dr. Omar Al Omari, on reasonable request.
